# Should development of Alzheimer’s disease-specific intravenous immunoglobulin be considered?

**DOI:** 10.1186/s12974-014-0198-z

**Published:** 2014-12-05

**Authors:** David A Loeffler

**Affiliations:** Department of Internal Medicine, Division of Neurology, Beaumont Health System, 3601 West Thirteen Mile Road, Royal Oak, MI 48073 USA

**Keywords:** Aβ, Alzheimer’s disease, Antibodies, Complement activation, Cytokines, Immunotherapy, Intravenous immunoglobulin

## Abstract

Recent phase II and III studies with intravenous immunoglobulin (IVIG) in patients with Alzheimer’s disease (AD) did not find evidence for the slowing of AD progression compared to placebo-treated patients, in contrast to encouraging results in pilot studies. An additional phase III trial is ongoing. If negative results are found, then further AD studies with IVIG are unlikely unless a manufacturer opts for a trial with high-dose IVIG, which would increase its anti-inflammatory effects but also the risk for adverse events. An alternative approach could be an AD-specific IVIG, supplementing IVIG with higher concentrations of selected antibodies purified from it or produced via recombinant polyclonal antibody technology. These antibodies could include those to amyloid-beta (Aβ, tau protein, inflammatory cytokines, complement activation proteins, and the receptor for advanced glycation end products. IgG fragment crystallizable (Fc) fragments containing terminal sialic acid could be added to increase anti-inflammatory effects. While this product might be more effective in slowing AD clinical progression than current IVIG, there are difficulties with this approach. Preclinical studies would be required to determine which of the antibodies of interest for supplementing current IVIG (for example, antibodies to phosphorylated or oligomeric tau) are actually present (and, therefore, available for purification) in IVIG, and the effects of the product in mouse models of AD. An Investigational New Drug application for an AD-specific IVIG would require United States Food and Drug Administration approval. If the drug would be found to benefit AD patients, meeting the increased demand for IVIG would be challenging.

## Review

### Introduction

Approximately 5.2 million Americans are currently diagnosed with Alzheimer’s disease (AD). The prevalence of this disorder in the United States is 4% for individuals under 65 years of age, 15% for those between 65 and 74 years of age, 44% for those between 75 and 84 years of age, and 38% for individuals 85 years of age and older [1]. Estimates of individuals with AD or other dementias worldwide range from 36 to nearly 44 million people [2,3].

The five drugs approved by the United States Food and Drug Administration for treating AD provide short-term symptomatic benefits to approximately half of the patients who receive them, but are not believed to influence neuropathological progression of the disease. Since the amyloid cascade hypothesis was published in 1991 [4], AD therapy has focused primarily on amyloid-beta (Aβ, but Aβ vaccination [5], monoclonal anti-Aβ antibodies [6-8], β-secretase inhibitors [9], and γ-secretase modulators [10] and inhibitors [11] have not succeeded in slowing the progressive loss of cognitive functioning that occurs in AD. Interest has increased in the targeting of tau pathology (neurofibrillary tangles (NFTs), dystrophic neurites, and neuropil threads) which correlates more strongly than plaque counts with AD’s cognitive deficits [12-14].

Intravenous immunoglobulin (IVIG) is another approach that has been examined for the treatment of AD. IVIG products contain purified plasma immunoglobulins (primarily IgG) from large numbers of healthy donors. These drugs are used to treat many autoimmune, immunodeficiency, and inflammatory disorders. The full range of antibodies in the human immune repertoire, estimated at 10^9^ [15], is thought to be present in IVIG. Among the antibodies in IVIG are naturally occurring auto-antibodies, which are produced against both self and altered self antigens in the absence of external antigen exposure (reviewed by Lutz *et al*. [16] and Schwartz-Albiez *et al*. [17]). Natural antibodies, which comprise approximately two-thirds of the human immune repertoire [18,19], are generated against intracellular constituents, plasma proteins, cell-surface antigens, and neoantigens [16]. Some of these antibodies, referred to as anti-idiotypic antibodies, can bind to variable regions of other antibodies, potentially interfering with the biological activities of these antibodies; with relevance to AD, IVIG was recently shown to contain Aβ anti-idiotypic antibodies (antibodies to its anti-Aβ antibodies [20]). IVIG’s anti-idiotypic antibodies have been suggested to be responsible for its beneficial effects in some autoimmune disorders [21,22].

Many natural antibodies are polyreactive, meaning they can bind to more than one antigen [23]. Octapharma’s IVIG Octagam™ was shown by Dodel *et al*. in 2002 to contain anti-Aβ antibodies [24], and these findings were subsequently replicated by others [25,26]. On this basis its effects, and then those of Baxter Healthcare’s Gammagard™, were examined in AD pilot studies [27,28] which found that IVIG could decrease cerebrospinal fluid Aβ and increase plasma Aβ, suggesting increased removal of Aβ from the brain. This was followed by a phase II study with Gammagard [29] which indicated that IVIG might stabilize or improve cognitive functioning in AD patients. Subsequent phase II and III trials with Octagam and Gammagard, respectively, gave disappointing results. In the Octagam study [30], there were no significant differences in cognitive and functional scores between any of the treatment groups and the placebo group. No significant differences were found between five of the six IVIG dose (treatment) groups and the placebo group for plasma Aβ1-40, and in the sixth treatment group, plasma Aβ1-40 decreased versus placebo. Cerebrospinal fluid (CSF) Aβ1-40 and Aβ1-42, and hippocampal and total brain volume were not influenced by IVIG treatment. In the Gammagard trial [31], no significant differences were detected between the IVIG and placebo groups for measures of cognitive functioning or activities of daily living, despite the lowering of brain levels of fibrillar Aβ1-42. Some benefits versus the placebo group with regard to cognitive scores were detected in Apolipoprotein E4^+^ AD patients and in patients with mild AD, but the trial was not powered to detect differences between subgroups. A phase III trial with Grifols’ IVIG Flebogamma™ is underway in which AD patients will undergo plasmapheresis and be treated with albumin and Flebogamma [32]. The effects of Sutter Health’s IVIG NewGam™ are also being examined in individuals with mild cognitive impairment (MCI) [33], thought to be the transitional state between the cognitive changes of normal aging and very early dementia [34].

Svetlicky *et al*. [35] reviewed the beneficial results that have been obtained when ‘target-specific’ IVIG (which they referred to as ‘sIVIG’) preparations have been used to treat experimental animal models of autoimmune diseases. These disorders include systemic lupus erythematosus [36], anti-phospholipid syndrome [37], myasthenia gravis [38], pemphigus vulgaris [39], and small-vessel vasculitis [35]. In each case, the specific IVIG preparation consisted of anti-idiotypic antibodies purified from IVIG, which targeted the antibody responsible for the autoimmune disorder in the animal model. A study was cited in which IVIG prepared from donors with high levels of antibodies to West Nile virus was used to successfully treat patients with West Nile virus encephalitis [40]. Another disease-specific IVIG is polyclonal anti-D, which is prepared from plasma from rhesus D (RhD)-negative donors immunized to the D antigen and used to treat patients with idiopathic thrombocytopenic purpura [41]. It was recently suggested that an AD-specific IVIG preparation could be more effective than current IVIG products for the treatment of AD [42].

### What options remain for intravenous immunoglobulin treatment of Alzheimer’s disease?

The doses of IVIG employed in the AD trials were those used for antibody replacement in immunodeficiency syndromes, so whether the higher doses which are required for IVIG’s anti-inflammatory effects (1 g/kg [43]) might be beneficial in AD is unknown [44]; an even higher dose, 2/g/kg, is required for efficient inhibition of complement activation [45]. Administration of high-dose IVIG has been associated with increased risk for thromboembolic complications, intravascular hemolysis, acute renal failure, and aseptic meningitis [46-48]. The study by Katz *et al*. [47] concluded that these problems can often be avoided by using slow infusion rates, maintaining adequate hydration, and avoiding patients with known risk factors such as advanced age, previous thromboembolic events, immobilization, diabetes mellitus, hypertension, and dyslipidemia. Unfortunately, most AD patients have one or more of these risk factors.

A second option could be an AD-specific IVIG. The effects of this product could initially be compared to those of current IVIG products in a mouse model of AD which develops both plaques and NFTs, such as the 3xTg-AD mouse [49]. Most IVIG studies in AD mouse models have been performed in mice which develop only Aβ pathology. These studies have found conflicting results [50-53]. Dodel *et al*. [54], using anti-Aβ antibodies purified from IVIG, reported reduced plaque counts in three-month-old, but not 12-month-old APP695 double-mutant mice. In 3xTg-AD mice IVIG treatment had no effect on plaque deposition or insoluble Aβ, although it decreased 56 kDa Aβ oligomers by 60%, and no influence on tau pathology [55]. A second IVIG study in this mouse model reported a 25 to 30% decrease in AT-180^+^ (hyperphosphorylated tau-containing) hippocampal CA1 neurons [56]. Evidence for increased neuroprotective or behavioral benefits of treatment with AD-specific IVIG, compared to standard IVIG, in 3xTg-AD mice would provide support for a pilot study to assess the safety of an AD-specific IVIG preparation in AD patients.

### What antibodies could be included in Alzheimer’s disease-specific intravenous immunoglobulin?

An AD-specific IVIG product could be generated by simply supplementing a current IVIG product with higher levels of its anti-Aβ antibodies, which have been shown to exert neuroprotective effects *in vitro* and in some mouse models of AD [50,54,57-59]. A second approach would be to combine other AD-relevant antibodies and terminally-sialylated fragment crystallizable (Fc) fragments, in addition to anti-Aβ antibodies, that would also be purified from IVIG. The extent to which the concentrations of each of these components should be increased, in comparison to their levels in current IVIG preparations, could be examined in mouse studies and perhaps later in an AD pilot study.

The few studies that have compared the levels of AD-related antibodies between IVIG products found differences between the products [25,26,60]. These differences are likely to be due to variations in production methods and/or plasma donor populations. There have been no studies comparing the effects of various IVIG products in AD patients, so whether one product would be preferable to another for the preparation of AD-specific IVIG is unknown.

A potential advantage of IVIG over monoclonal antibodies for AD therapy is that it contains antibodies against multiple proteins that are thought to contribute to AD’s development and progression. However, IVIG’s polyvalent antibodies have a range of antigen-binding affinities [61]. An AD-specific IVIG might be more effective if the antibodies to be added to current IVIG possess at least moderate antigen-binding affinity. In practice, this would require using only the affinity-purified antibodies from later elution fractions, rather than pooling all of the eluted antibody fractions. AD-specific IVIG could be produced by supplementing a current IVIG product with some, or all, of the following antibodies:

### Anti-Amyloid-beta (Aβ) antibodies

Some studies have reported that IVIG’s anti-Aβ antibodies are limited to those that are ‘conformation-specific’ (they do not recognize linear Aβ) [62], while others suggest that they may bind to monomeric Aβ, as well as Aβ aggregates [26,54,58]. Aβ25-40 is a major region for IVIG binding, while its binding to Aβ’s N-terminus is minimal [25]. Phase III trials with two monoclonal anti-Aβ antibodies, Bapineuzumab and Solanezumab, which were generated against linear N-terminal and central-domain Aβ epitopes respectively, failed to slow the decline of cognitive functioning in AD patients [6,7], although in the Solanezumab trial some benefits to patients with mild AD were detected. More recently a phase II trial with another anti-Aβ monoclonal, Crenezumab, also produced negative results, although benefits were again observed in the mild AD group [8]. Crenezumab was generated against Aβ12-23 and bound to Aβ monomer, oligomers, and fibrils [63]. Because the degradation of fibrillar Aβ, including by anti-Aβ antibodies, might shift the distribution of Aβ aggregates from fibrils to more neurotoxic Aβ oligomers [64], optimally the purified anti-Aβ antibodies to be used for supplementing current IVIG products should be specific for Aβ soluble oligomers, although this may not be possible. Of relevance is a recent study [65] in which repeated administration of monoclonal sequence-independent anti-oligomer antibodies to 3xTg-AD mice resulted in improved cognitive performance, reduced hippocampal plaques and brain levels of soluble and insoluble Aβ40 and Aβ42, and reduced microglial activation. This treatment also reduced tau hyperphosphorylation in hippocampal CA1 neurons, providing additional evidence for a relationship between AD’s Aβ and tau pathology [66-68]. Aβ oligomers have been suggested to induce activation of the enzyme glycogen synthase kinase-3β (GSK-3β), which is thought to contribute to tau’s hyperphosphorylation in the AD brain [69].

### Anti-tau antibodies

Tau is an intraneuronal protein located primarily in axons, where it plays an important role in microtubule formation and stabilization through its binding to tubulin [70]. The extent of its phosphorylation controls its microtubule binding. Phosphorylation of normal tau is limited to two or three of its 441 amino acids, but when its hyperphosphorylation (phosphorylation of more than 39 of tau’s amino acids) occurs in AD [71] its binding to microtubules decreases, resulting in reduced axonal transport and subsequent neuronal injury. The significance of NFTs in AD is unresolved; although NFT counts and distribution in the brain are positively correlated with AD’s cognitive deficits [12,13,72], NFT-bearing neurons can survive for decades [73] so NFTs are not acutely neurotoxic. Hyperphosphorylation of tau, together with its aggregation, leads to formation of the paired helical filaments (PHF) found in NFTs [74], so both processes are potential targets for AD therapy. Administration of antibodies specific for phosphorylated tau epitopes reduced tau pathology in transgenic NFT-bearing mice [75-77], and a tau aggregation inhibitor slowed AD’s progression in a phase II trial [78]. A phase I trial to examine the safety of a vaccine targeting misfolded tau protein in AD patients is in the recruiting phase [79].

IVIG products contain anti-tau antibodies [60], including some which bind specifically to its microtubule-binding domains (MBD) [80], which are thought to be required for tau’s aggregation [81,82]. Antibodies to tau’s MBD could be included in AD-specific IVIG, based on the finding that monoclonal antibodies against these regions have been shown to reduce tau’s aggregation [83]. The neuroprotective effects of IVIG’s anti-tau antibodies, and whether some of these antibodies are specific for pathogenic tau conformations (phosphorylated or aggregated tau), are unknown. A recent study by Counts *et al*. [56] showed that IVIG treatment of 3xTg-AD mice reduced hippocampal CA1 tau pathology; however, this result could have been due to IVIG’s anti-tau antibodies and/or its anti-Aβ antibodies. Rosenmann *et al*. [84] reported the presence of serum antibodies in healthy aged controls and AD patients to a phosphorylated tau fragment (tau 195-213, phosphorylated at tau 202/205), suggesting that such antibodies may be present in IVIG (and therefore be available for purification, and subsequent supplementation of these antibodies, in AD-specific IVIG). However, in that study, binding of serum antibodies to non-phosphorylated tau 195-213 was apparently not examined, so the possibility that the serum immunoglobulin binding could have been to non-phosphorylated rather than phosphorylated tau cannot be ruled out.

### Antibodies to inflammatory cytokines (or inclusion of terminally sialylated Fc fragments as an alternative)

There is an extensive literature about increased inflammatory processes in the AD brain (reviewed by Akiyama *et al*., [85] and Mrak and Griffin [86]). Central nervous system (CNS) inflammation may be a prerequisite for developing AD [87]. Anti-cytokine antibodies reported in IVIG include those to interleukin-1α (IL-1α) and interleukin-6 (IL-6) [88], interferon-α (IFN-α) and interferon-β (IFN-β) [89], interferon-γ (IFN-γ) [90], granulocyte macrophage-colony stimulating factor (GM-CSF) [91], BAFF (B-cell Activating Factor of the tumor necrosis factor (TNF) Family), and APRIL (A PRoliferation-Inducing Ligand) [92]. IL-1α, IL-6, and GM-CSF are inflammatory cytokines [93-95], while IFN-α and IFN-γ have both pro- and anti-inflammatory actions [96,97] and IFN-β is anti-inflammatory [98]. Treatment of the Tg2576 AD mouse model with anti-GM-CSF antibody decreased microglial activation and brain Aβ deposition [99], but, paradoxically, administration of GM-CSF reversed cognitive impairment and amyloidosis in transgenic AD mice [100], so whether AD-specific IVIG should be supplemented with IVIG’s anti-GM-CSF antibodies is unclear. The safest approach might be to limit the anti-cytokine antibodies used to supplement IVIG in an AD-specific preparation to those targeting IL-1α and IL-6. The necessity for including even these antibodies is debatable because the anti-inflammatory effects of IVIG have been reported to be fully accounted for by its IgG Fc fragments (specifically, terminal sialic acid residues on the Asn297-linked glycan of IVIG, which is present on just 1 to 2% of its IgG molecules [101,102]). On this basis, Nimmerjahn and Ravetch [101] proposed that a sialic acid-enriched IVIG product could be prepared which would confer greater anti-inflammatory activity at far lower IVIG doses than those currently required to generate this activity. Therefore AD-specific IVIG could be supplemented with sialylated Fc fragments obtained from IVIG. Of relevance is a study by Käsermann *et al*. [103], in which sialic acid-enriched IgG fractions were obtained from IVIG. Sialic acid residues were identified in both Fab and Fc fragments. Surprisingly, anti-inflammatory activity was associated only with sialylated Fab. IVIG may also reduce inflammation by inducing the release of interleukin-10 [104] and IL-1 receptor antagonist (IL-1ra) [105], both of which have anti-inflammatory actions [106].

### Anti-complement antibodies

IVIG inhibits cell surface deposition of early complement activation fragments, the opsonins C4b and C3b [107]. It binds to the anaphylatoxins C3a and C5a to neutralize their pro-inflammatory effects [108], and reduces the concentration of C3b2-containing complexes [109], despite the fact that IVIG can moderately activate complement [110]. C3b2 complexes are composed of two C3b molecules linked to IgG or another plasma protein, and function as efficient C3 convertase precursors [109,111]. IVIG also prevents development and deposition of C5b-9, the membrane attack complex (MAC) [112], which is neurotoxic [113] and present on AD plaques and NFTs [114]. IVIG’s ability to prevent complement-mediated neuronal cell death was demonstrated in a mouse stroke model [115]. The role of complement activation in AD is complex and its significance is unclear, so whether IVIG’s neuroprotective effects in AD would be helped or harmed by increasing its anti-complement activities is uncertain. Deposition of C4b and C3b on pathogenic conformations of Aβ (neuritic plaques) or tau (extracellular (‘tombstone’) tangles) should increase their removal by activated microglia, so if IVIG inhibits deposition of these opsonins on Aβ or tau, it could impair the ability of the CNS immune response to remove them. However, reduction of MAC formation by IVIG should be beneficial. Conflicting results as to the relationship of complement activation to AD-type pathology have been obtained in studies with mouse transgenic models of AD [116-120]. Interpretation of these studies is complicated by the fact that complement is not activated to the same extent in the mouse AD models as in AD [121,122]. IVIG administration was shown to protect against synaptic dysfunction in the Tg2576 mouse model of AD by increasing brain levels of C5a [123], but the factor in IVIG responsible for this activity was not identified. A better understanding of IVIG’s antibodies to complement activation proteins, and the effects of these antibodies, is needed to determine which, if any, of these antibodies should be supplemented in AD-specific IVIG.

### Anti-receptor for advanced glycation end products antibodies

Advanced glycation end products (AGE) form when reducing sugars react with amino groups in proteins, lipids, and amino acids [124]. The receptor for AGE (RAGE) is present on microglia, neurons, and the blood-brain barrier (BBB) [125], and RAGE gene expression is increased in AD hippocampal pyramidal cells, cortical neurons, and glia [126]. Activation of microglial RAGE causes release of free radicals and inflammatory cytokines [127], inducing cytotoxic effects, while RAGE’s presence on the BBB allows it to transport Aβ from peripheral blood into the brain [128]. The interaction between RAGE and Aβ at the BBB can lead to oxidative stress, inflammatory responses, and reduced cerebral blood flow [129]. RAGE is a therapeutic target of interest in AD because its blockade reduces neuronal and synaptic injury [130]. The anti-RAGE antibodies reportedly present in IVIG [131] could block the receptor, reducing the influx of Aβ into the brain, and thereby lowering brain Aβ levels [19]. It would be appropriate to include these antibodies in AD-specific IVIG.

### Other antibodies

IVIG has additional neuroprotective actions, including antioxidant [132] and anti-apoptotic [133] effects. The latter study found that IVIG treatment reduced Aβ-induced neuronal p38MAPK, a kinase involved in cell death mechanisms, including apoptosis and excitotoxicity, which can be activated by Aβ [134]. The antibodies in IVIG which were responsible for these effects are unknown. Apoptosis is reported to be increased in the AD brain [135,136], and Fas (CD95), a member of the TNF receptor superfamily, can initiate this process [137]. Its binding by the cell surface molecule Fas ligand (Fas-L) can induce apoptosis. Anti-Fas antibodies can act as Fas agonists to trigger apoptosis [138,139] or they can function as neutralizing antibodies to prevent cell death from occurring via the Fas-L pathway [140]. Both apoptotic [141] and anti-apoptotic [133,142] effects have been reported for IVIG, which contains anti-Fas antibodies [143]. Which of these actions IVIG exerts may depend on the dose of IVIG [144]. Until more is known about the actions of IVIG’s anti-Fas antibodies, they should not be included in AD-specific IVIG.

### Potential difficulties with Alzheimer’s disease-specific intravenous immunoglobulin

Treatment with IVIG can raise serum viscosity, increasing the risk for thromboembolic events [145]. However, given the large number of antibodies in IVIG, supplementing it with increased levels of a few selected antibodies and possibly also Fc fragments should not appreciably increase its osmolality. Because this would be a new drug, approval of an Investigative New Drug (IND) application by the United States Food and Drug Administration would be required.

Even if an AD-specific IVIG would be found to exert beneficial effects in AD patients, its relatively short duration of action would require continued treatment, which would consume a great deal of IVIG. The subjects in the IVIG AD trials received IVIG every two or four weeks [27,28,30] and a similar regimen would likely be required for AD-specific IVIG. Neuroprotective effects would not be expected to be maintained if administration of AD-specific IVIG would be discontinued; in the AD pilot study with Gammagard performed by Relkin *et al*. [28], mini-mental state scores in AD patients increased by an average of 2.5 points after six months of treatment, but returned to baseline after a three-month washout period (the half-life of IgG in IVIG was reported to be 25.8 days, similar to endogenous IgG [146]). Further, if AD-specific IVIG would benefit AD patients, these effects might be seen only in mild-to-moderate AD patients, but not in individuals in which the disease had progressed further. In the phase III AD trial with Gammagard, one of the two subgroups for which ‘favorable cognitive changes’ were reported was AD patients with moderate impairments [31].

With the exception of IVIG’s anti-Aβ antibodies [54], the effects of its other antibodies discussed above have not been examined using purified antibodies in mouse models of AD. IVIG manufacturers will need to decide if the effects of each of these antibodies should be evaluated separately in a mouse model of AD before determining which of the antibodies to include in AD-specific IVIG.

The anti-tau antibodies which were reported in IVIG [60] bound to full-length recombinant human tau (tau 1-441). The possibility is not ruled out that if the levels of these antibodies are increased in AD-specific IVIG, the resulting preparation could induce an autoimmune response. Although such an outcome has not been reported following IVIG treatment, Rosenmann *et al*. [147] found that vaccination of C57BL/6 mice with full-length human tau induced anti-tau antibodies which were associated with development of tau pathology and encephalitogenicity (manifested by neurologic deficits). Two injections consisting of tau emulsified in complete Freund’s adjuvant (CFA) were given one week apart. Pertussis toxin, which can induce a transient increase in BBB permeability [148], was given the same day as the first injection and 48 hours later. The immunopotentiating effects of CFA, combined with BBB impairment due to pertussis toxin, may have contributed to the autoimmune neuropathy which developed in these animals. Rosenmann *et al*. subsequently reported the development of paralytic disease in wild-type mice and ‘tauopathy’ mice that were repeatedly immunized with a mixture of three phosphorylated tau fragments emulsified in CFA and pertussis toxin [149]. However, they found no evidence for encephalitogenicity when only one injection of the CFA and pertussis-emulsified phosphorylated tau was given, followed by the phosphorylated tau fragments alone one week later [150]. The rationale for emulsifying phosphorylated tau with CFA and pertussis toxin in these studies was that the pro-inflammatory environment induced by this protocol in the CNS might more closely model the situation in AD patients, perhaps allowing for a better assessment of the potential risks of immunizing AD patients with phosphorylated tau than could be obtained using less inflammation-promoting adjuvants. Studies in tauopathy mice in which anti-phospho-tau antibodies were administered [75-77] found no autoimmune problems.

Regulatory issues would be of concern with regard to United States Food and Drug Administration approval of an AD-specific IVIG. There is a precedent for United States Food and Drug Administration approval of a multi-component plasma product: Factor Eight Inhibitor Bypassing Activity (FEIBA, produced by Baxter Healthcare) is an activated prothrombin complex concentrate which, similar to IVIG, is produced from pooled human plasma. It contains four coagulation factors, namely factors II, VII, IX, and X [151,152]. FEIBA is used for the treatment of hemophilia patients with inhibitors (inhibitory antibodies produced secondary to treatment with clotting factors). It has been used therapeutically for more than 35 years (reviewed by Cromwell and Aledort [153]).

If studies in mouse AD models suggest increased benefits of AD-specific IVIG compared to standard IVIG, then consideration could be given to producing the antibodies required to supplement current IVIG through recombinant technology, rather than purifying them from IVIG. The technology is available for production of recombinant human polyclonal antibodies on an industrial scale [154,155]. This approach would assure lot-to-lot uniformity for each of the antibodies and would reduce the amount of IVIG required to produce AD-specific IVIG.

A final obstacle would be the economic one. The question of how adequate supplies of IVIG could be maintained if there is an increased demand for it in AD patients was discussed previously [156] and will be briefly summarized here. Processing of a plasma sample into IVIG requires about nine months [157], so the supply of IVIG cannot be rapidly replenished in the event of a shortage of the product. Extensive use of AD-specific IVIG would reduce the IVIG available for individuals with severe primary immunodeficiencies, for whom IVIG is the standard of care [158]. Further, IVIG treatment is expensive, with costs for IVIG treatment of the 5.4 million Americans with AD estimated to be $280 billion per year [159]. Based upon an IVIG price of $75 per gram, the cost to administer 0.4 g/kg of IVIG (a dose used in AD trials) to an 80 kg individual every other week for a year would be $62,400. This would be prohibitive for many AD patients.

## Conclusions

Although the multiple antibodies in IVIG should give it an advantage over monoclonal antibody for treatment of AD, its encouraging results in pilot studies have not been replicated in larger trials. Administration of high-dose IVIG would increase its anti-inflammatory effects but would likely be associated with increased adverse events, and many AD patients have risk factors which could preclude their receiving high-dose IVIG. An alternative approach could be to develop an AD-specific IVIG. This would require additional time and expense, but it might be the last and best chance for IVIG treatment of AD to succeed.

## Abbreviations

AD: Alzheimer’s disease
AGE: Advanced glycation end products
APRIL: A proliferation-inducing ligand
BAFF: B-cell activating factor of the tumor necrosis factor family
BBB: Blood-brain barrier
CFA: Complete Freund’s adjuvant
CNS: central nervous sytem
CSF: cerebrospinal fluid
Fas-L: Fas ligand
Fc: fragment crystallizable (fragment of IgG)
FEIBA: Factor eight inhibitor bypassing activity
GM-CSF: Granulocyte macrophage-colony stimulating factor
GSK-3β: Glycogen synthase kinase-3β
IL-1α: Interleukin-1α
IL-1ra: IL-1 receptor antagonist
IL-6: Interleukin-6
IFN-α: Interferon-α
IFN-β: Interferon-β
IFN- γ: Interferon-γ
IVIG: Intravenous immunoglobulin
MAC: Membrane attack complex
MBD: Microtubule binding domains
MCI: Mild cognitive impairment
NFTs: Neurofibrillary tangles
sIVIG: Target-specific IVIG
RAGE: Receptor for AGE
RhD: Rhesus D
TNF: Tumor necrosis factor

## References

[1] Association A’s (2014). Alzheimer’s disease facts and figures. Alzheimers Dement.

[2] Alzheimers.net: **Alzheimer’s statistics.** [http://www.alzheimers.net/resources/alzheimers-statistics/]

[3] BrightFocus Foundation: **Alzheimer’s facts & statistics.** [http://www.brightfocus.org/alzheimers/about/understanding/facts.html]

[4] Hardy J, Allsop D (1991). Amyloid deposition as the central event in the aetiology of Alzheimer’s disease. Trends Pharmacol Sci.

[5] Gilman S, Koller M, Black RS, Jenkins L, Griffith SG, Fox NC, Eisner L, Kirby L, Rovira MB, Forette F, Orgogozo JM, AN1792(QS-21)-201 Study Team (2005). Clinical effects of abeta immunization (AN1792) in patients with AD in an interrupted trial. Neurology.

[6] Salloway S, Sperling R, Gilman S, Fox NC, Blennow K, Raskind M, Sabbagh M, Honig LS, Doody R, van Dyck CH, Mulnard R, Barakos J, Gregg KM, Liu E, Lieberburg I, Schenk D, Black R, Grundman M, Bapineuzumab 201 Clinical Trial Investigators (2009). A phase 2 multiple ascending dose trial of bapineuzumab in mild to moderate Alzheimer disease. Neurology.

[7] Lilly: **Lilly announces detailed results of the phase 3 solanezumab expedition studies following a presentation of the independent analyses by the Alzheimer’s Disease Cooperative Study (ADCS).** [https://investor.lilly.com/releasedetail.cfm?releaseid=711933]

[8] Roche: **Roche announces phase II clinical results of crenezumab in Alzheimer’s disease.** [http://www.roche.com/investors/ir_update/inv-update-2014-07-16.htm]

[9] Lilly: **Lilly voluntarily terminates phase II study for LY2886721, a beta secretase inhibitor, being investigated as a treatment for Alzheimer’s disease.** [https://investor.lilly.com/releasedetail.cfm?ReleaseID=771353]

[10] Green RC, Schneider LS, Amato DA, Beelen AP, Wilcock G, Swabb EA, Zavitz KH (2009). Tarenflurbil phase 3 study group: effect of tarenflurbil on cognitive decline and activities of daily living in patients with mild Alzheimer disease: a randomized controlled trial. JAMA.

[11] Lilly: **Lilly halts development of semagacestat for Alzheimer’s disease based on preliminary results of phase III clinical trials.** In [https://investor.lilly.com/releasedetail.cfm?releaseid=499794]

[12] Markesbery WR (1997). Neuropathological criteria for the diagnosis of Alzheimer’s disease. Neurobiol Aging.

[13] Haroutunian V, Purohit DP, Perl DP, Marin D, Khan K, Lantz M, Davis KL, Mohs RC (1999). Neurofibrillary tangles in nondemented elderly subjects and mild Alzheimer disease. Arch Neurol.

[14] Giannakopoulos P, Herrmann FR, Bussière T, Bouras C, Kövari E, Perl DP, Morrison JH, Gold G, Hof PR (2003). Tangle and neuron numbers, but not amyloid load, predict cognitive status in Alzheimer’s disease. Neurology.

[15] Bayry J, Kazatchkine MD, Kaveri SV (2007). Shortage of human intravenous immunoglobulin – reasons and possible solutions. Nat Clin Pract Neurol.

[16] Lutz HU, Binder CJ, Kaveri S (2009). Naturally occurring auto-antibodies in homeostasis and disease. Trends Immunol.

[17] Schwartz-Albiez R, Monteiro RC, Rodriguez M, Binder CJ, Shoenfeld Y (2009). Natural antibodies, intravenous immunoglobulin and their role in autoimmunity, cancer and inflammation. Clin Exp Immunol.

[18] Shoenfeld Y, Gershwin ME, Meroni PL (2007). Autoantibodies (Second Edition).

[19] Dodel R, Neff F, Noelker C, Pul R, Du Y, Bacher M, Oertel W (2010). Intravenous immunoglobulins as a treatment for Alzheimer’s disease: rationale and current evidence. Drugs.

[20] Loeffler DA, Klaver AC, Coffey MP: **Aβ anti-idiotypic antibodies are present in intravenous immunoglobulin and are produced in mice following its administration.** *Autoimmunity* 2014, Nov 13:1-5. [Epub ahead of print]. doi:10.3109/08916934.2014.983265. 10.3109/08916934.2014.983265 25392130

[21] Kazatchkine MD, Kaveri SV (2001). Immunomodulation of autoimmune and inflammatory diseases with intravenous immune globulin. N Engl J Med.

[22] Sapir T, Blank M, Shoenfeld Y (2005). Immunomodulatory effects of intravenous immunoglobulins as a treatment for autoimmune diseases, cancer, and recurrent pregnancy loss. Ann N Y Acad Sci.

[23] Avrameas S (1991). Natural autoantibodies: from ‘horror autotoxicus’ to ‘gnothi seauton’. Immunol Today.

[24] Dodel R, Hampel H, Depboylu C, Lin S, Gao F, Schock S, Jäckel S, Wei X, Buerger K, Höft C, Hemmer B, Möller HJ, Farlow M, Oertel WH, Sommer N, Du Y (2002). Human antibodies against amyloid beta peptide: a potential treatment for Alzheimer’s disease. Ann Neurol.

[25] Balakrishnan K, Andrei-Selmer LC, Selmer T, Bacher M, Dodel R (2010). Comparison of intravenous immunoglobulins for naturally occurring autoantibodies against amyloid-beta. J Alzheimers Dis.

[26] Klaver AC, Finke JM, Digambaranath J, Balasubramaniam M, Loeffler DA (2010). Antibody concentrations to Abeta1-42 monomer and soluble oligomers in untreated and antibody-antigen-dissociated intravenous immunoglobulin preparations. Int Immunopharmacol.

[27] Dodel RC, Du Y, Depboylu C, Hampel H, Frölich L, Haag A, Hemmeter U, Paulsen S, Teipel SJ, Brettschneider S, Spottke A, Nölker C, Möller HJ, Wei X, Farlow M, Sommer N, Oertel WH (2004). Intravenous immunoglobulins containing antibodies against beta-amyloid for the treatment of Alzheimer’s disease. J Neurol Neurosurg Psychiatry.

[28] Relkin NR, Szabo P, Adamiak B, Burgut T, Monthe C, Lent RW, Younkin S, Younkin L, Schiff R, Weksler ME (2009). 18-Month study of intravenous immunoglobulin for treatment of mild Alzheimer disease. Neurobiol Aging.

[29] Relkin N, Bettger L, Tsakanikas D, Ravdin L (2012). Three-year follow-up on the IVIG for Alzheimer’s phase II study. Alzheimers Dement.

[30] Dodel R, Rominger A, Bartenstein P, Barkhof F, Blennow K, Förster S, Winter Y, Bach JP, Popp J, Alferink J, Wiltfang J, Buerger K, Otto M, Antuono P, Jacoby M, Richter R, Stevens J, Melamed I, Goldstein J, Haag S, Wietek S, Farlow M, Jessen F (2013). Intravenous immunoglobulin for treatment of mild-to-moderate Alzheimer’s disease: a phase 2, randomised, double-blind, placebo-controlled, dose-finding trial. Lancet Neurol.

[31] Alzforum: **Gammagard™ misses endpoints in phase 3 trial.** [http://www.alzforum.org/news/research-news/gammagardtm-misses-endpoints-phase-3-trial]

[32] ClinicalTrials.gov: **A study to evaluate albumin and immunoglobulin in Alzheimer’s disease (AMBAR).** In [https://clinicaltrials.gov/show/NCT01561053]

[33] ClinicalTrials.gov: **Study of intravenous immunoglobulin in amnestic Mild Cognitive Impairment (MCI).** [http://www.clinicaltrials.gov/ct2/show/NCT01300728]

[34] Petersen RC, Negash S (2008). Mild cognitive impairment: an overview. CNS Spectr.

[35] Svetlicky N, Ortega-Hernandez OD, Mouthon L, Guillevin L, Thiesen HJ, Altman A, Kravitz MS, Blank M, Shoenfeld Y (2013). The advantage of specific intravenous immunoglobulin (sIVIG) on regular IVIG: experience of the last decade. J Clin Immunol.

[36] Shoenfeld Y, Rauova L, Gilburd B, Kvapil F, Goldberg I, Kopolovic J, Rovensky J, Blank M (2002). Efficacy of IVIG affinity-purified anti-double-stranded DNA anti-idiotypic antibodies in the treatment of an experimental murine model of systemic lupus erythematosus. Int Immunol.

[37] Blank M, Anafi L, Zandman-Goddard G, Krause I, Goldman S, Shalev E, Cervera R, Font J, Fridkin M, Thiesen HJ, Shoenfeld Y (2007). The efficacy of specific IVIG anti-idiotypic antibodies in antiphospholipid syndrome (APS): trophoblast invasiveness and APS animal model. Int Immunol.

[38] Fuchs S, Feferman T, Meidler R, Margalit R, Sicsic C, Wang N, Zhu KY, Brenner T, Laub O, Souroujon MC (2008). A disease-specific fraction isolated from IVIG is essential for the immunosuppressive effect of IVIG in experimental autoimmune myasthenia gravis. J Neuroimmunol.

[39] Mimouni D, Blank M, Payne AS, Anhalt GJ, Avivi C, Barshack I, David M, Shoenfeld Y (2010). Efficacy of intravenous immunoglobulin (IVIG) affinity-purified anti-desmoglein anti-idiotypic antibodies in the treatment of an experimental model of pemphigus vulgaris. Clin Exp Immunol.

[40] Makhoul B, Braun E, Herskovitz M, Ramadan R, Hadad S, Norberto K (2009). Hyperimmune gammaglobulin for the treatment of West Nile virus encephalitis. Isr Med Assoc J.

[41] Salama A, Kiefel V, Mueller-Eckhardt C (1986). Effect of IgG anti-Rho(D) in adult patients with chronic autoimmune thrombocytopenia. Am J Hematol.

[42] Knight EM, Gandy S (2014). Immunomodulation and AD–down but not out. J Clin Immunol.

[43] Kaneko Y, Nimmerjahn F, Ravetch JV (2006). Anti-inflammatory activity of immunoglobulin G resulting from Fc sialylation. Science.

[44] Relkin N (2014). Clinical trials of intravenous immunoglobulin for Alzheimer’s disease. J Clin Immunol.

[45] Hack CE, Scheltens P (2004). Intravenous immunoglobulins: a treatment for Alzheimer’s disease?. J Neurol Neurosurg Psychiatry.

[46] Brannagan TH (2002). Intravenous gammaglobulin (IVIg) for treatment of CIDP and related immune-mediated neuropathies. Neurology.

[47] Katz U, Achiron A, Sherer Y, Shoenfeld Y (2007). Safety of intravenous immunoglobulin (IVIG) therapy. Autoimmun Rev.

[48] Welles CC, Tambra S, Lafayette RA (2010). Hemoglobinuria and acute kidney injury requiring hemodialysis following intravenous immunoglobulin infusion. Am J Kidney Dis.

[49] Oddo S, Caccamo A, Kitazawa M, Tseng BP, LaFerla FM (2003). Amyloid deposition precedes tangle formation in a triple transgenic model of Alzheimer’s disease. Neurobiol Aging.

[50] Magga J, Puli L, Pihlaja R, Kanninen K, Neulamaa S, Malm T, Härtig W, Grosche J, Goldsteins G, Tanila H, Koistinaho J, Koistinaho M (2010). Human intravenous immunoglobulin provides protection against Aβ toxicity by multiple mechanisms in a mouse model of Alzheimer’s disease. J Neuroinflammation.

[51] Puli L, Pomeshchik Y, Olas K, Malm T, Koistinaho J, Tanila H (2012). Effects of human intravenous immunoglobulin on amyloid pathology and neuroinflammation in a mouse model of Alzheimer’s disease. J Neuroinflammation.

[52] Gong B, Pan Y, Zhao W, Knable L, Vempati P, Begum S, Ho L, Wang J, Yemul S, Barnum S, Bilski A, Gong BY, Pasinetti GM (2013). IVIG immunotherapy protects against synaptic dysfunction in Alzheimer’s disease through complement anaphylatoxin C5a-mediated AMPA-CREB-C/EBP signaling pathway. Mol Immunol.

[53] Sudduth TL, Greenstein A, Wilcock DM (2013). Intracranial injection of Gammagard, a human IVIg, modulates the inflammatory response of the brain and lowers Aβ in APP/PS1 mice along a different time course than anti-Aβ antibodies. J Neurosci.

[54] Dodel R, Balakrishnan K, Keyvani K, Deuster O, Neff F, Andrei-Selmer LC, Röskam S, Stüer C, Al-Abed Y, Noelker C, Balzer-Geldsetzer M, Oertel W, Du Y, Bacher M (2011). Naturally occurring autoantibodies against beta-amyloid: investigating their role in transgenic animal and in vitro models of Alzheimer’s disease. J Neurosci.

[55] St-Amour I, Paré I, Tremblay C, Coulombe K, Bazin R, Calon F (2014). IVIg protects the 3xTg-AD mouse model of Alzheimer’s disease from memory deficit and Aβ pathology. J Neuroinflammation.

[56] Counts S, Perez S, He B, Mufson E (2014). IVIG reduces tau pathology and preserves neuroplastic gene expression in the 3xTg mouse model of Alzheimer’s disease. Curr Alz Res.

[57] Istrin G, Bosis E, Solomon B (2006). Intravenous immunoglobulin enhances the clearance of fibrillar amyloid-beta peptide. J Neurosci Res.

[58] Szabo P, Relkin N, Weksler ME (2008). Natural human antibodies to amyloid beta peptide. Autoimmun Rev.

[59] Du Y, Wei X, Dodel R, Sommer N, Hampel H, Gao F, Ma Z, Zhao L, Oertel WH, Farlow M (2003). Human anti-beta-amyloid antibodies block beta-amyloid fibril formation and prevent beta-amyloid-induced neurotoxicity. Brain.

[60] Smith LM, Coffey MP, Klaver AC, Loeffler DA (2013). Intravenous immunoglobulin products contain specific antibodies to recombinant human tau protein. Int Immunopharmacol.

[61] Szabo P, Mujalli DM, Rotondi ML, Sharma R, Weber A, Schwarz HP, Weksler ME, Relkin N (2010). Measurement of anti-beta amyloid antibodies in human blood. J Neuroimmunol.

[62] Welzel AT, Williams AD, McWilliams-Koeppen HP, Acero L, Weber A, Blinder V, Mably A, Bunk S, Hermann C, Farrell MA, Ehrlich HJ, Schwarz HP, Walsh DM, Solomon A, O’Nuallain B (2012). Human anti-Aβ IgGs target conformational epitopes on synthetic dimer assemblies and the AD brain-derived peptide. PLoS One.

[63] Adolfsson O, Pihlgren M, Toni N, Varisco Y, Buccarello AL, Antoniello K, Lohmann S, Piorkowska K, Gafner V, Atwal JK, Maloney J, Chen M, Gogineni A, Weimer RM, Mortensen DL, Friesenhahn M, Ho C, Paul R, Pfeifer A, Muhs A, Watts RJ (2012). An effector-reduced anti-β-amyloid (Aβ) antibody with unique aβ binding properties promotes neuroprotection and glial engulfment of Aβ. J Neurosci.

[64] Cohen FE, Kelly JW (2003). Therapeutic approaches to protein-misfolding diseases. Nature.

[65] Rasool S, Martinez-Coria H, Wu JW, LaFerla F, Glabe CG (2013). Systemic vaccination with anti-oligomeric monoclonal antibodies improves cognitive function by reducing Aβ deposition and tau pathology in 3xTg-AD mice. J Neurochem.

[66] Oddo S, Caccamo A, Tran L, Lambert MP, Glabe CG, Klein WL, LaFerla FM (2006). Temporal profile of amyloid-beta (Abeta) oligomerization in an in vivo model of Alzheimer disease. a link between Abeta and tau pathology. J Biol Chem.

[67] Fein JA, Sokolow S, Miller CA, Vinters HV, Yang F, Cole GM, Gylys KH (2008). Co-localization of amyloid beta and tau pathology in Alzheimer’s disease synaptosomes. Am J Pathol.

[68] Wray S, Noble W (2009). Linking amyloid and tau pathology in Alzheimer’s disease: the role of membrane cholesterol in Abeta-mediated tau toxicity. J Neurosci.

[69] Ma QL, Lim GP, Harris-White ME, Yang F, Ambegaokar SS, Ubeda OJ, Glabe CG, Teter B, Frautschy SA, Cole GM (2006). Antibodies against beta-amyloid reduce Abeta oligomers, glycogen synthase kinase-3beta activation and tau phosphorylation in vivo and in vitro. J Neurosci Res.

[70] Weingarten MD, Lockwood AH, Hwo SY, Kirschner MW (1975). A protein factor essential for microtubule assembly. Proc Natl Acad Sci U S A.

[71] Hanger DP, Byers HL, Wray S, Leung KY, Saxton MJ, Seereeram A, Reynolds CH, Ward MA, Anderton BH (2007). Novel phosphorylation sites in tau from Alzheimer brain support a role for casein kinase 1 in disease pathogenesis. J Biol Chem.

[72] Bierer LM, Hof PR, Purohit DP, Carlin L, Schmeidler J, Davis KL, Perl DP (1995). Neocortical neurofibrillary tangles correlate with dementia severity in Alzheimer’s disease. Arch Neurol.

[73] Morsch R, Simon W, Coleman PD (1999). Neurons may live for decades with neurofibrillary tangles. J Neuropathol Exp Neurol.

[74] Rankin CA, Sun Q, Gamblin TC (2007). Tau phosphorylation by GSK-3beta promotes tangle-like filament morphology. Mol Neurodegener.

[75] Sigurdsson EM (2008). Immunotherapy targeting pathological tau protein in Alzheimer’s disease and related tauopathies. J Alzheimers Dis.

[76] Chai X, Wu S, Murray TK, Kinley R, Cella CV, Sims H, Buckner N, Hanmer J, Davies P, O’Neill MJ, Hutton ML, Citron M (2011). Passive immunization with anti-Tau antibodies in two transgenic models: reduction of Tau pathology and delay of disease progression. J Biol Chem.

[77] Boutajangout A, Ingadottir J, Davies P, Sigurdsson EM (2011). Passive immunization targeting pathological phospho-tau protein in a mouse model reduces functional decline and clears tau aggregates from the brain. J Neurochem.

[78] Wischik CM, Bentham P, Wischik DJ, Seng KM (2008). Tau aggregation inhibitor (TAI) therapy with remberTM arrests disease progression in mild and moderate Alzheimer’s disease over 50 weeks. Alzheimers and Dementia.

[79] ClinicalTrials.gov: **Safety study of AADvac1, a Tau Peptide-KLH-conjugate active vaccine to treat Alzheimer’s disease.** [http://clinicaltrials.gov/show/NCT01850238]

[80] Smith LM, Coffey MP, Loeffler DA (2014). Specific binding of intravenous immunoglobulin products to tau peptide fragments. Int Immunopharmacol.

[81] von Bergen M, Friedhoff P, Biernat J, Heberle J, Mandelkow EM, Mandelkow E (2000). Assembly of tau protein into Alzheimer paired helical filaments depends on a local sequence motif ((306)VQIVYK(311)) forming beta structure. Proc Natl Acad Sci U S A.

[82] Tokimasa M, Minoura K, Hiraoka S, Tomoo K, Sumida M, Taniguchi T, Ishida T (2005). Importance of local structures of second and third repeat fragments of microtubule-binding domain for tau filament formation. FEBS Lett.

[83] Taniguchi T, Sumida M, Hiraoka S, Tomoo K, Kakehi T, Minoura K, Sugiyama S, Inaka K, Ishida T, Saito N, Tanaka C (2005). Effects of different anti-tau antibodies on tau fibrillogenesis: RTA-1 and RTA-2 counteract tau aggregation. FEBS Lett.

[84] Rosenmann H, Meiner Z, Geylis V, Abramsky O, Steinitz M (2006). Detection of circulating antibodies against tau protein in its unphosphorylated and in its neurofibrillary tangles-related phosphorylated state in Alzheimer’s disease and healthy subjects. Neurosci Lett.

[85] Akiyama H, Barger S, Barnum S, Bradt B, Bauer J, Cole GM, Cooper NR, Eikelenboom P, Emmerling M, Fiebich BL, Finch CE, Frautschy S, Griffin WS, Hampel H, Hull M, Landreth G, Lue L, Mrak R, Mackenzie IR, McGeer PL, O’Banion MK, Pachter J, Pasinetti G, Plata-Salaman C, Rogers J, Rydel R, Shen Y, Streit W, Strohmeyer R, Tooyoma I (2000). Inflammation and Alzheimer’s disease. Neurobiol Aging.

[86] Mrak RE, Griffin WS (2001). Interleukin-1, neuroinflammation, and Alzheimer’s disease. Neurobiol Aging.

[87] Lue LF, Brachova L, Civin WH, Rogers J (1996). Inflammation, a beta deposition, and neurofibrillary tangle formation as correlates of Alzheimer’s disease neurodegeneration. J Neuropathol Exp Neurol.

[88] Svenson M, Hansen MB, Bendtzen K (1993). Binding of cytokines to pharmaceutically prepared human immunoglobulin. J Clin Invest.

[89] Ross C, Svenson M, Hansen MB, Vejlsgaard GL, Bendtzen K (1995). High avidity IFN-neutralizing antibodies in pharmaceutically prepared human IgG. J Clin Invest.

[90] Toungouz M, Denys C, Dupont E (1996). Blockade of proliferation and tumor necrosis factor-alpha production occurring during mixed lymphocyte reaction by interferon-gamma-specific natural antibodies contained in intravenous immunoglobulins. Transplantation.

[91] Svenson M, Hansen MB, Ross C, Diamant M, Rieneck K, Nielsen H, Bendtzen K (1998). Antibody to granulocyte-macrophage colony-stimulating factor is a dominant anti-cytokine activity in human IgG preparations. Blood.

[92] Le Pottier L, Sapir T, Bendaoud B, Youinou P, Shoenfeld Y, Pers JO (2007). Intravenous immunoglobulin and cytokines: focus on tumor necrosis factor family members BAFF and APRIL. Ann N Y Acad Sci.

[93] Dinarello CA (1991). Inflammatory cytokines: interleukin-1 and tumor necrosis factor as effector molecules in autoimmune diseases. Curr Opin Immunol.

[94] Ershler WB (1993). Interleukin-6: a cytokine for gerontologists. J Am Geriatr Soc.

[95] Zhan Y, Vega-Ramos J, Carrington EM, Villadangos JA, Lew AM, Xu Y (2012). The inflammatory cytokine, GM-CSF, alters the developmental outcome of murine dendritic cells. Eur J Immunol.

[96] Tilg H, Peschel C (1996). Interferon-alpha and its effects on the cytokine cascade: a pro- and anti-inflammatory cytokine. Leuk Lymphoma.

[97] Mühl H, Pfeilschifter J (2003). Anti-inflammatory properties of pro-inflammatory interferon-gamma. Int Immunopharmacol.

[98] Cakebread JA, Xu Y, Grainge C, Kehagia V, Howarth PH, Holgate ST, Davies DE (2011). Exogenous IFN-β has antiviral and anti-inflammatory properties in primary bronchial epithelial cells from asthmatic subjects exposed to rhinovirus. J Allergy Clin Immunol.

[99] Manczak M, Mao P, Nakamura K, Bebbington C, Park B, Reddy PH (2009). Neutralization of granulocyte macrophage colony-stimulating factor decreases amyloid beta 1-42 and suppresses microglial activity in a transgenic mouse model of Alzheimer’s disease. Hum Mol Genet.

[100] Boyd TD, Bennett SP, Mori T, Governatori N, Runfeldt M, Norden M, Padmanabhan J, Neame P, Wefes I, Sanchez-Ramos J, Arendash GW, Potter H (2010). GM-CSF upregulated in rheumatoid arthritis reverses cognitive impairment and amyloidosis in Alzheimer mice. J Alzheimers Dis.

[101] Nimmerjahn F, Ravetch JV (2007). The antiinflammatory activity of IgG: the intravenous IgG paradox. J Exp Med.

[102] Anthony RM, Nimmerjahn F, Ashline DJ, Reinhold VN, Paulson JC, Ravetch JV (2008). Recapitulation of IVIG anti-inflammatory activity with a recombinant IgG Fc. Science.

[103] Käsermann F, Boerema DJ, Rüegsegger M, Hofmann A, Wymann S, Zuercher AW, Miescher S (2012). Analysis and functional consequences of increased Fab-sialylation of intravenous immunoglobulin (IVIG) after lectin fractionation. PLoS One.

[104] Lories RJ, Casteels-Van Daele M, Ceuppens JL, Van Gool SW (2004). Polyclonal immunoglobulins for intravenous use induce interleukin 10 release in vivo and in vitro. Ann Rheum Dis.

[105] Craciun LI, DiGiambattista M, Laub R, Goldman M, Dupont E (2007). Apoptosis: a target for potentiation of UV-induced IL-1Ra synthesis by IVIg. Immunol Lett.

[106] Wolf AM, Wolf D, Rumpold H, Enrich B, Tilg H (2004). Adiponectin induces the anti-inflammatory cytokines IL-10 and IL-1RA in human leukocytes. Biochem Biophys Res Commun.

[107] Basta M, Fries LF, Frank MM (1991). High doses of intravenous Ig inhibit in vitro uptake of C4 fragments onto sensitized erythrocytes. Blood.

[108] Basta M, Van Goor F, Luccioli S, Billings EM, Vortmeyer AO, Baranyi L, Szebeni J, Alving CR, Carroll MC, Berkower I, Stojilkovic SS, Metcalfe DD (2003). F(ab)'2-mediated neutralization of C3a and C5a anaphylatoxins: a novel effector function of immunoglobulins. Nat Med.

[109] Lutz HU, Stammler P, Bianchi V, Trüeb RM, Hunziker T, Burger R, Jelezarova E, Späth PJ (2004). Intravenously applied IgG stimulates complement attenuation in a complement-dependent autoimmune disease at the amplifying C3 convertase level. Blood.

[110] Wegmüller E (1998). Effect of intravenous immunoglobulin therapy on plasma complement. Transfus Sci.

[111] Lutz HU, Stammler P, Jelezarova E, Nater M, Späth PJ (1996). High doses of immunoglobulin G attenuate immune aggregate-mediated complement activation by enhancing physiologic cleavage of C3b in C3bn-IgG complexes. Blood.

[112] Basta M, Dalakas MC (1994). High-dose intravenous immunoglobulin exerts its beneficial effect in patients with dermatomyositis by blocking endomysial deposition of activated complement fragments. J Clin Invest.

[113] van Beek J, Elward K, Gasque P (2003). Activation of complement in the central nervous system: roles in neurodegeneration and neuroprotection. Ann N Y Acad Sci.

[114] Webster S, Lue LF, Brachova L, Tenner AJ, McGeer PL, Terai K, Walker DG, Bradt B, Cooper NR, Rogers J (1997). Molecular and cellular characterization of the membrane attack complex, C5b-9, in Alzheimer’s disease. Neurobiol Aging.

[115] Arumugam TV, Tang SC, Lathia JD, Cheng A, Mughal MR, Chigurupati S, Magnus T, Chan SL, Jo DG, Ouyang X, Fairlie DP, Granger DN, Vortmeyer A, Basta M, Mattson MP (2007). Intravenous immunoglobulin (IVIG) protects the brain against experimental stroke by preventing complement-mediated neuronal cell death. Proc Natl Acad Sci U S A.

[116] Wyss-Coray T, Yan F, Lin AH, Lambris JD, Alexander JJ, Quigg RJ, Masliah E (2002). Prominent neurodegeneration and increased plaque formation in complement-inhibited Alzheimer’s mice. Proc Natl Acad Sci U S A.

[117] Zhou J, Fonseca MI, Pisalyaput K, Tenner AJ (2008). Complement C3 and C4 expression in C1q sufficient and deficient mouse models of Alzheimer’s disease. J Neurochem.

[118] Maier M, Peng Y, Jiang L, Seabrook TJ, Carroll MC, Lemere CA (2008). Complement C3 deficiency leads to accelerated amyloid beta plaque deposition and neurodegeneration and modulation of the microglia/macrophage phenotype in amyloid precursor protein transgenic mice. J Neurosci.

[119] Ager RR, Fonseca MI, Chu SH, Sanderson SD, Taylor SM, Woodruff TM, Tenner AJ (2010). Microglial C5aR (CD88) expression correlates with amyloid-beta deposition in murine models of Alzheimer’s disease. J Neurochem.

[120] Fonseca MI, Chu SH, Berci AM, Benoit ME, Peters DG, Kimura Y, Tenner AJ (2011). Contribution of complement activation pathways to neuropathology differs among mouse models of Alzheimer’s disease. J Neuroinflammation.

[121] Schwab C, Hosokawa M, McGeer PL (2004). Transgenic mice overexpressing amyloid beta protein are an incomplete model of Alzheimer disease. Exp Neurol.

[122] Reichwald J, Danner S, Wiederhold KH, Staufenbiel M (2009). Expression of complement system components during aging and amyloid deposition in APP transgenic mice. J Neuroinflammation.

[123] Gong B, Levine S, Barnum SR, Pasinetti GM (2014). Role of complement systems in IVIG mediated attenuation of cognitive deterioration in Alzheimer's disease. Curr Alzheimer Res.

[124] Singh R, Barden A, Mori T, Beilin L (2001). Advanced glycation end-products: a review. Diabetologia.

[125] Schmidt AM, Sahagan B, Nelson RB, Selmer J, Rothlein R, Bell JM (2009). The role of RAGE in amyloid-beta peptide-mediated pathology in Alzheimer’s disease. Curr Opin Investig Drugs.

[126] Hudson BI, Stickland MH, Grant PJ (1998). Identification of polymorphisms in the receptor for advanced glycation end products (RAGE) gene: prevalence in type 2 diabetes and ethnic groups. Diabetes.

[127] Berbaum K, Shanmugam K, Stuchbury G, Wiede F, Körner H, Münch G (2008). Induction of novel cytokines and chemokines by advanced glycation endproducts determined with a cytometric bead array. Cytokine.

[128] Deane R, Du Yan S, Submamaryan RK, LaRue B, Jovanovic S, Hogg E, Welch D, Manness L, Lin C, Yu J, Zhu H, Ghiso J, Frangione B, Stern A, Schmidt AM, Armstrong DL, Arnold B, Liliensiek B, Nawroth P, Hofman F, Kindy M, Stern D, Zlokovic B (2003). RAGE mediates amyloid-beta peptide transport across the blood-brain barrier and accumulation in brain. Nat Med.

[129] Deane RJ (2012). Is RAGE still a therapeutic target for Aleimer’s disease?. Future Med Chem.

[130] Yan SS, Chen D, Yan S, Guo L, Du H, Chen JX (2012). RAGE is a key cellular target for Abeta-induced perturbation in Alzheimer’s disease. Front Biosci (Schol Ed).

[131] Weber A, Engelmaier A, Teschner W, Ehrlich HJ, Schwarz HP (2009). Intravenous immunoglobulin (IVIg) Gammagard liquid contains anti-RAGE IgG and sLRP. Alzheimers Dement.

[132] Counts SE, Ray B, Mufson EJ, Perez SE, He B, Lahiri DK (2014). Intravenous immunoglobulin (IVIG) treatment exerts antioxidant and neuropreservatory effects in preclinical models of Alzheimer’s disease. J Clin Immunol.

[133] Widiapradja A, Vegh V, Lok KZ, Manzanero S, Thundyil J, Gelderblom M, Cheng YL, Pavlovski D, Tang SC, Jo DG, Magnus T, Chan SL, Sobey CG, Reutens D, Basta M, Mattson MP, Arumugam TV (2012). Intravenous immunoglobulin protects neurons against amyloid beta-peptide toxicity and ischemic stroke by attenuating multiple cell death pathways. J Neurochem.

[134] Yang ZH, Sun K, Suo WH, Yao LY, Fu Q, Cui YY, Fu GH, Chen HZ, Lu Y (2010). N-stearoyltyrosine protects primary neurons from Aβ-induced apoptosis through modulating mitogen-activated protein kinase activity. Neuroscience.

[135] Overmyer M, Kraszpulski M, Helisalmi S, Soininen H, Alafuzoff I (2000). DNA fragmentation, gliosis and histological hallmarks of Alzheimer’s disease. Acta Neuropathol.

[136] Cribbs DH, Poon WW, Rissman RA, Blurton-Jones M (2004). Caspase-mediated degeneration in Alzheimer’s disease. Am J Pathol.

[137] Kaufmann T, Strasser A, Jost PJ (2012). Fas death receptor signalling: roles of Bid and XIAP. Cell Death Differ.

[138] Komada Y, Sakurai M (1997). Fas receptor (CD95)-mediated apoptosis in leukemic cells. Leuk Lymphoma.

[139] Chodorge M, Züger S, Stirnimann C, Briand C, Jermutus L, Grütter MG, Minter RR (2012). A series of Fas receptor agonist antibodies that demonstrate an inverse correlation between affinity and potency. Cell Death Differ.

[140] Uslu R, Borsellino N, Frost P, Gárban H, Ng CP, Mizutani Y, Belldegrun A, Bonavida B (1997). Chemosensitization of human prostate carcinoma cell lines to anti-fas-mediated cytotoxicity and apoptosis. Clin Cancer Res.

[141] Nakatani K, Takeshita S, Tsujimoto H, Sekine I (2002). Intravenous immunoglobulin (IVIG) preparations induce apoptosis in TNF-alpha-stimulated endothelial cells via a mitochondria-dependent pathway. Clin Exp Immunol.

[142] Arredondo J, Chernyavsky AI, Karaouni A, Grando SA (2005). Novel mechanisms of target cell death and survival and of therapeutic action of IVIg in Pemphigus. Am J Pathol.

[143] Reipert BM, Stellamor MT, Poell M, Ilas J, Sasgary M, Reipert S, Zimmermann K, Ehrlich H, Schwarz HP (2008). Variation of anti-Fas antibodies in different lots of intravenous immunoglobulin. Vox Sang.

[144] Altznauer F, von Gunten S, Späth P, Simon HU (2003). Concurrent presence of agonistic and antagonistic anti-CD95 autoantibodies in intravenous Ig preparations. J Allergy Clin Immunol.

[145] Dalakas MC (1994). High-dose intravenous immunoglobulin and serum viscosity: risk of precipitating thromboembolic events. Neurology.

[146] Mankarious S, Lee M, Fischer S, Pyun KH, Ochs HD, Oxelius VA, Wedgwood RJ (1988). The half-lives of IgG subclasses and specific antibodies in patients with primary immunodeficiency who are receiving intravenously administered immunoglobulin. J Lab Clin Med.

[147] Rosenmann H, Grigoriadis N, Karussis D, Boimel M, Touloumi O, Ovadia H, Abramsky O (2006). Tauopathy-like abnormalities and neurologic deficits in mice immunized with neuronal tau protein. Arch Neurol.

[148] Kügler S, Böcker K, Heusipp G, Greune L, Kim KS, Schmidt MA (2007). Pertussis toxin transiently affects barrier integrity, organelle organization and transmigration of monocytes in a human brain microvascular endothelial cell barrier model. Cell Microbiol.

[149] Rozenstein-Tsalkovich L, Grigoriadis N, Lourbopoulos A, Nousiopoulou E, Kassis I, Abramsky O, Karussis D, Rosenmann H (2013). Repeated immunization of mice with phosphorylated-tau peptides causes neuroinflammation. Exp Neurol.

[150] Boimel M, Grigoriadis N, Lourbopoulos A, Haber E, Abramsky O, Rosenmann H (2010). Efficacy and safety of immunization with phosphorylated tau against neurofibrillary tangles in mice. Exp Neurol.

[151] Collins PW (2012). Therapeutic challenges in acquired factor VIII deficiency. Hematol Am Soc Hematol Educ Program.

[152] Awad N, Cocchio C (2013). Activated prothrombin complex concentrates for the reversal of anticoagulant-associated coagulopathy. Proc Natl Acad Sci U S A.

[153] Cromwell C, Aledort LM (2012). FEIBA: a prohemostatic agent. Semin Thromb Hemost.

[154] Wiberg FC, Rasmussen SK, Frandsen TP, Rasmussen LK, Tengbjerg K, Coljee VW, Sharon J, Yang CY, Bregenholt S, Nielsen LS, Haurum JS, Tolstrup AB (2006). Production of target-specific recombinant human polyclonal antibodies in mammalian cells. Biotechnol Bioeng.

[155] Rasmussen SK, Rasmussen LK, Weilguny D, Tolstrup AB (2007). Manufacture of recombinant polyclonal antibodies. Biotechnol Lett.

[156] Loeffler DA (2013). Intravenous immunoglobulin and Alzheimer’s disease: what now?. J Neuroinflammation.

[157] McFalls K: **Plasma therapies: where are we now?** [http://www.fffenterprises.com/news/articles/article-2011-05-03.html]

[158] Shehata N, Palda V, Bowen T, Haddad E, Issekutz TB, Mazer B, Schellenberg R, Warrington R, Easton D, Anderson D, Hume H (2010). The use of immunoglobulin therapy for patients with primary immune deficiency: an evidence-based practice guideline. Transfus Med Rev.

[159] Mintz M: **What if IVIG really worked for Alzheimer’s?** [http://drmintz.blogspot.com/2012/07/what-if-ivig-really-worked-for.html]

