# Erratum: Should development of Alzheimer’s disease-specific intravenous immunoglobulin be considered?

**DOI:** 10.1186/s12974-015-0290-z

**Published:** 2015-04-09

**Authors:** David A Loeffler

**Affiliations:** Department of Internal Medicine, Division of Neurology, Beaumont Health System, 3601 West Thirteen Mile Road, Royal Oak, 48073 MI USA

## Erratum

After publication of our article [1], it came to our attention that we had erroneously stated that the IVIG product NewGam was produced by Sutter Health. However, Octapharma is the manufacturer of NewGamTM and Sutter Health is the sponsor of the clinical trial. Therefore the following sentence on page 2 of the original manuscript [1] is incorrect: "The effects of Sutter Health's IVIG NewGamTM are also being examined in individuals with mild cognitive impairment (MCI) [33], thought to be the transitional state between the cognitive changes of normal aging and very early dementia [34]." This sentence should read as follows: “The effects of Octapharma's IVIG NewGamTM are also being examined in individuals with mild cognitive impairment (MCI) [33], thought to be the transitional state between the cognitive changes of normal aging and very early dementia [34].”
